# Ursodeoxycholic and chenodeoxycholic bile acids attenuate systemic and liver inflammation induced by lipopolysaccharide in rats

**DOI:** 10.1007/s11010-024-04994-2

**Published:** 2024-04-05

**Authors:** T Milivojac, M Grabež, A Krivokuća, U Maličević, M Gajić Bojić, Đ Đukanović, S Uletilović, N Mandić-Kovačević, T Cvjetković, M Barudžija, N Vojinović, A Šmitran, Lj Amidžić, MP Stojiljković, M Čolić, M Mikov, R Škrbić

**Affiliations:** 1 Centre for Biomedical Research, Faculty of Medicine, University of Banja Luka, The Republic of Srpska, Banja Luka, Bosnia and Herzegovina; 2 Department of Hygiene, Faculty of Medicine, University of Banja Luka, The Republic of Srpska, Banja Luka, Bosnia and Herzegovina; 3 Department of Pathophysiology, Faculty of Medicine, University of Banja Luka, The Republic of Srpska, Banja Luka, Bosnia and Herzegovina; 4 Department of Pharmacology, Toxicology and Clinical Pharmacology, Faculty of Medicine, University of Banja Luka, The Republic of Srpska, Banja Luka, Bosnia and Herzegovina; 5 Department of Pharmacy, Faculty of Medicine, University of Banja Luka, The Republic of Srpska, Banja Luka, Bosnia and Herzegovina; 6 Department of Medical Biochemistry and Chemistry, Faculty of Medicine, The Republic of Srpska, University of Banja Luka, Banja Luka, Bosnia and Herzegovina; 7 Department of Histology and Embryology, Faculty of Medicine, The Republic of Srpska, University of Banja Luka, Banja Luka, Bosnia and Herzegovina; 8 Department of Microbiology and Immunology, Faculty of Medicine, The Republic of Srpska, University of Banja Luka, Banja Luka, Bosnia and Herzegovina; 9 Medical Faculty Foča, University of East Sarajevo, The Republic of Srpska, Banja Luka, Bosnia and Herzegovina; 10 Serbian Academy of Sciences and Arts, Belgrade, Serbia; 11 Department of Pharmacology, Toxicology and Clinical Pharmacology, Faculty of Medicine, University of Novi Sad, Novi Sad, Serbia

**Keywords:** Chenodeoxycholic acid, Ursodeoxycholic acid, Lipopolysaccharide, Endotoxemia, Oxidative stress, Inflammation

## Abstract

Bacterial lipopolysaccharide (LPS) induces general inflammation, by activating pathways involving cytokine production, blood coagulation, complement system activation, and acute phase protein release. The key cellular players are leukocytes and endothelial cells, that lead to tissue injury and organ failure. The aim of this study was to explore the anti-inflammatory, antioxidant, and cytoprotective properties of two bile acids, ursodeoxycholic acid (UDCA) and chenodeoxycholic acid (CDCA) in LPS-induced endotoxemia in rats. The experiment involved six distinct groups of Wistar rats, each subjected to different pretreatment conditions: control and LPS groups were pretreated with propylene glycol, as a bile acid solvent, while the other groups were pretreated with UDCA or CDCA for 10 days followed by an LPS injection on day 10. The results showed that both UDCA and CDCA reduced the production of pro-inflammatory cytokines: TNF-α, GM-CSF, IL-2, IFNγ, IL-6, and IL-1β and expression of nuclear factor-κB (NF-κB) induced by LPS. In addition, pretreatment with these bile acids showed a positive impact on lipid profiles, a decrease in ICAM levels, an increase in antioxidant activity (SOD, |CAT, GSH), and a decrease in prooxidant markers (H_2_O_2_ and O_2_^–^). Furthermore, both bile acids alleviated LPS-induced liver injury. While UDCA and CDCA pretreatment attenuated homocysteine levels in LPS-treated rats, only UDCA pretreatment showed reductions in other serum biochemical markers, including creatine kinase, lactate dehydrogenase, and high-sensitivity troponin I. It can be concluded that both, UDCA and CDCA, although exerted slightly different effects, can prevent the inflammatory responses induced by LPS, improve oxidative stress status, and attenuate LPS-induced liver injury.

## Introduction

Bacterial lipopolysaccharide (LPS), commonly referred to as endotoxin, is an important constituent of Gram-negative bacteria cell walls that is vital for bacterial cell viability, integrity and defense against environmental stress. It triggers the activation of the immune system and the onset of an acute systemic inflammatory response. The effects of LPS are mediated by a pathogen recognition receptor known as the Toll-like receptor 4 (TLR4) that is present on the surface of phagocytic cells such as macrophages, neutrophils, and dendritic cells. The activation of TLR4 initiates a signaling cascade that induces inflammation and production of pro-inflammatory cytokines [1]. This response can lead to the progressive dysfunction of two or more organ systems, known as multiple organ dysfunction syndrome (MODS), which represents the most severe form of the adverse course of sepsis and significantly contributes to mortality [2, 3]. Various pathophysiological mechanisms play a role in causing organ failure, including oxidative stress, inflammation, necrosis, and apoptosis [4]. Given the high mortality rate, it is imperative to initiate a range of studies to gain a deeper understanding of the fundamental pathophysiological mechanisms and develop novel therapeutic approaches for this complex syndrome.

A novel therapeutic approach, rooted in the role of bile acids, could potentially yield beneficial outcomes. For many years, bile acids were thought to possess only the emulsifying properties of dietary fats. However, increasing evidence suggests that bile acids also function as signaling molecules that regulate metabolism and immunity through the activation of specific receptors including the G protein-coupled bile acid receptor 1 (GPBAR1) or Takeda G-Protein receptor 5 (TGR5) and the Farnesoid-X receptor (FXR) [5, 6]. These receptors are involved in the host immune system and highly represented in cells of innate immunity such as macrophages, dendritic cells, and natural killer T cells originating from the intestinal system and liver [7]. The secondary bile acid, ursodeoxycholic acid (UDCA), generated through the conversion of the primary bile acid chenodeoxycholic acid (CDCA) in the gastrointestinal tract under the influence of gut microflora, offers a broad range of protective effects with minimal adverse effects [8]. In addition to studies highlighting cytoprotective and antiapoptotic effects [9, 10], numerous investigations emphasized the remarkable anti-inflammatory [11–13], immunomodulatory [6, 14], and antioxidant properties of UDCA [15, 16]. UDCA has been shown to inhibit endothelial dysfunction by mitigating endoplasmic reticulum stress [17], reducing the production of reactive oxygen species (ROS) [17–19], preventing the release of various pro-inflammatory cytokines, promoting the secretion of anti-inflammatory cytokines, and inhibiting the expression of nuclear factor κB (NF-κB) and inducible nitric oxide synthase (iNOS) induced by inflammation [19, 20].

Although CDCA has been associated with comparable effects [21–23], certain studies have hinted at its potential pro-inflammatory role in promoting liver injury [24]. It was also shown that CDCA increased IL-6, TNF-α, and vascular endothelial growth factor release in the intestinal mucosa of patients suffering from irritable bowel disease [25]. Controversies exist regarding the cytoprotective or cytotoxic effects of UDCA and CDCA, which act on different receptors and signalling molecules. We assume that pretreatment with these bile acids can significantly modulate the levels of inflammatory, endothelial, and oxidative stress markers in LPS-induced endotoxemia in rats. Having that in mind, the objective of this study was to compare the effects of two structurally different bile acids, UDCA and CDCA, on inflammatory and oxidative stress parameters and liver injury in endotoxemia induced by LPS.

## Materials and methods

### Experimental animals

Male Wistar albino rats (*n* = 36) were kept in individual cages (6 per cage) under controlled laboratory conditions (room temperature 21 ± 2 °C, 12-h light/dark cycle, and humidity 55 ± 5%) with free access to food and water. Rats were allowed to adapt to the environment for one week before the experiments. Experimental animals, protocols, and procedures were approved by the Ethics Committee for the Protection of Welfare of Experimental Animals of the Faculty of Medicine, University of Banja Luka (number 18/1.190-1/22, dated 9 March 2022).

### Experimental groups

The animals were divided into six groups, each consisting of six animals. They were pretreated with propylene glycol, UDCA, or CDCA for 10 days. The Control (C) group received 0.5 mL/kg of propylene glycol as a solvent per os (p.o.) for 10 days and 1mL/kg of saline intraperitoneally (i.p.) on the 10^th^ day. The LPS group received 0.5 mL/kg propylene glycol p.o. for 10 days and 5.5 mg/kg of LPS i.p on the 10^th^ day. The UDCA group was administrated 25 mg/kg of UDCA p.o. for 10 days and 1mL/kg of saline i.p. on the 10^th^ day. Rats in the UDCA + LPS group were pretreated with UDCA in the same manner as the UDCA group, but on the 10^th^ day, they received 5.5 mg/kg of LPS i.p. The CDCA group was given 25 mg/kg of CDCA p.o. for 10 days and 1mL/kg of saline i.p. on the 10^th^ day. Rats in the CDCA + LPS group were pretreated with CDCA in the same way as the CDCA group, but on the 10^th^ day, they received 5.5 mg/kg of LPS i.p.

The study was divided into two parts. The first part of the study was aimed at investigating the anti-inflammatory effects of 10-day-pretreatment with UDCA and CDCA before the administration of a nonlethal yet inflammatory dose of LPS. To assess the production of pro-inflammatory cytokines: TNF-α, granulocyte-macrophage colony-stimulating factor (GM-CSF), interleukin−2 (IL-2), interferon γ (IFNγ), IL-6, and interleukin-1β (IL-1β) the rats were sacrificed two hours after administration of LPS.

The second part of this study aimed to explore the impact of UDCA and CDCA pretreatment on oxidative stress and biochemical parameters, and liver injury in endotoxemia induced by LPS, and the rats were sacrificed 48 h after administration of the same dose of LPS. In addition, immunohistochemistry analyses were performed to determine the NF-κB expression in hepatocytes.

### Blood and tissue collection

At the end of the experiment, rats were anaesthetized using a combination of ketamine (90 mg/kg) and xylazine (10 mg/kg), and blood samples were collected from the aorta. The red blood cell lysate was obtained after separating plasma. Isolated red blood cells were washed three times in three volumes of cold saline. Plasma samples were centrifuged at 3,000 rpm for 10 min. Serum was obtained by allowing the blood samples to sit at room temperature for 20 min until a clot was formed, and then they were centrifuged for 5 min at 3,000 rpm. All samples were stored at -80 °C until measurement. The livers were isolated and placed in plastic containers with 10% formalin for immunohistochemical and histological analysis.

### Cytokine quantification

The plasma levels of TNF-α, GM-CSF, IL-2, IFNγ, IL-6, and IL-1β were determined by using the AimPlex Custom Rat 10 Plex Panel fluorescence-based microbeads (AimPlex Biosciences, Pomona, CA, USA) and flow cytometry (BD Biosciences, Franklin Lakes, NJ, USA). The soluble intercellular adhesion molecule 1 (ICAM 1) concentrations in the serum were determined by ELISA using a rat ICAM-1 (FineTest, Wuhan, China). Both assays were performed by following the manufacturer’s recommended protocol. The concentrations of investigated biomolecules were determined according to the known concentrations of analytes in the standards.

### Serum markers

The concentration of homocysteine (Hcy), high-sensitivity troponin I (hsTnI), triglycerides (TG), total cholesterol (TC), high-density lipoprotein (HDL-C) cholesterol, low-density lipoprotein (LDL-C) cholesterol, glucose, the activity of lactate dehydrogenase (LDH), alanine aminotransferase (ALT), aspartate aminotransferase (AST) and creatine kinase (CK) were measured by methods manufactured by Abbot Diagnostics on the Alinity c- analyzer (Abbot Laboratories, IL, USA).

### Oxidative stress markers

CAT and SOD activity and GSH levels were analyzed by Beutler methods [26–28] in red blood cell lysates and measured spectrophotometrically. Plasma H_2_O_2_ level was measured by the Pick and Keisari method, based on the H_2_O_2_ oxidation of phenol red, at a wavelength of 610 nm [29]. The plasma concentrations of O_2_^-^ were determined using Nitro Blue Tetrazolium (NTB) and TRIS buffer, and a wavelength of 530 nm was used for measurement [30].

### Immunohistochemical analysis

Immunohistochemical analysis was conducted on 3–4 µm deparaffinized and rehydrated tissue sections. The slides underwent a 20-minute boiling process in a PT Module (Thermo Scientific) with a citric acid buffer solution (0.01 mol/L citrate buffer, pH 6.0). To minimize the nonspecific background staining, slides were treated with 3% hydrogen peroxide for 10 minutes. The primary rabbit polyclonal antibody targeting NF-κB/p65 (dilution 1:100, Abcam, ab16502) was applied following the manufacturer’s recommended protocol. Phosphate-buffered saline (pH 7.4) was used for washing between the steps. Chromogenic 3,3’-Diaminobenzidine (DAB) (TL-015-HDJ, Thermo Scientific Lab Vision UltraVision ONE Detection System) was employed to develop the antigen-antibody complex. Subsequently, all slides were counterstained with hematoxylin and eosin (H&E), dehydrated, and mounted. Simultaneously, appropriate positive and negative controls underwent processing. Microscopic analysis (Leica DM 6000 B) was performed at 400 × magnification. Twenty non-successive fields per sample were assessed for the number of positive cells expressing NF-κB/p65 in a blinded manner, utilizing ImageJ software (National Institute of Health, Bethesda, MD, United States). The percentage of immunopositive hepatocytes for NF-κB/p65 was calculated as follows: % of positively stained cells = (Number of positively stained cells × 100) / Total number of cells.

### Histopathological analysis

The livers were extracted and preserved in buffered formalin, followed by the creation of tissue blocks using paraffin wax. Subsequently, each block was sliced into 4 μm sections of a microtome and then stained with H&E. To assess histological changes indicative of liver injuries, we assigned scores ranging from 1 to 4. Hepatic damage was semi-quantitatively evaluated in a low-power field, considering the severity and percentage of tissue damage. We utilized a grading system (0–4) where 0 indicated normal tissue, 1 represented less than 24% tissue injury, 2 denoted 26–50% tissue damage, 3 indicated tissue damage in the range of 51–75%, and 4 signified tissue damage exceeding 75%. The assessment criteria used by two independent pathologists in a blinded manner, included the examination of liver lobule architecture; evaluation of vascular congestion, hemorrhage, and extravasation; determination of hepatocyte necrosis, and the presence of inflammatory cells to evaluate the pathological aspects,

### Statistical analysis

Statistical analysis was performed with IBM-SPSS Statistic version 20.0 software (SPSS, Inc., Chicago IL, USA). ANOVA test is used to compare the means of parametric characteristics and Kruskal-Wallis and Mann-Whitney U tests are used to compare the nonparametric characteristics between the groups. Tukey and Bonferroni tests are used for *post hoc* analysis. Results are presented as mean ± standard error, and the level of significance was set at p ˂ 0.05.

## Results

### Effects of UDCA and CDCA pretreatment on pro-inflammatory cytokines production two hours after administration of LPS

The concentrations of of TNF-α, GM-CSF, IL-2, IFNγ, IL-6, and IL-1β were significantly increased 2 h upon challenge of non-lethal dose of LPS (*p* < .01, *p* < .001, *p* < .01, *p* < .05, *p* < .01, *p* < .05, respectively). Both, UDCA and CDCA pretreatment given orally for 10 days exerted anti-inflammatory effects manifested by a significant decrease in the levels of TNF-α, GM-CSF, and IL-1β in the LPS-induced endotoxemia. (*p* < .05). Only pretreatment with UDCA led to a significant decrease in the level of IL-6 (*p* < .05), while pretreatment with CDCA only led to a significant decrease in the level of IFN-γ (*p* < .01) (Table 1).

**Table 1:** Effects of pretreatment of UDCA and CDCA on pro-inflammatory cytokines concentration measured 2 h after LPS administration

Parameters	Groups						
	C	LPS		UDCA	UDCA + LPS	CDCA	CDCA + LPS
TNF-α (pg/mL)	4.48 ± 2.06	19.41 ± 8.01††	3.91 ± 0.64		9.96 ± 1.02*	4.69 ± 2.12	6.36 ± 3.84**
GM-CSF (pg/mL)	9.57 ± 19.46	179.66 ± 55.82†††	6.67 ± 4.49		85.24 ± 64.95*	3.95 ± 2.96	106.60 ± 57.87*
IL-2 (pg/mL)	1.88 ± 1.69	18.82 ± 13.23††	5.16 ± 5.08		10.70 ± 6.28	6.54 ± 4.44	8.38 ± 2.55
IFN-γ (pg/mL)	6.97 ± 5.63	14.52 ± 5.90†	13.36 ± 3.85		10.73 ± 2.10	8.53 ± 2.75	6.41 ± 1.60**
IL-6 (pg/mL)	24.50 ± 15.76	64.85 ± 13.42††	28.52 ± 22.66		43.83 ± 15.23*	46.56 ± 17.19	44.70 ± 23.51
IL-1β (pg/mL)	2.96 ± 2.19	25.11 ± 22.67†	1.61 ± 1.07		2.19 ± 1–58*	5.43 ± 3.21	1.67 ± 0.2*

C- control group; LPS- LPS treated group; UDCA- Ursodeoxycholic acid treated group; UDCA + LPS- Ursodeoxycholic acid and LPS treated group; CDCA- Chenodeoxycholic acid treated group; CDCA + LPS- Chenodeoxycholic acid and LPS treated group. Data are expressed as mean ± SD (*n* = 6). Mann-Whitney test and asterisk (*) indicate significant differences compared with LPS group * *p* < .05, ** *p* < .01 *** *p* < .001, and sign (†) indicate significant differences compared with C group †† *p* < .01, ††† *p* < .001

### Effects of UDCA and CDCA pretreatment on body weight (BW), liver weight (LW), and LW/BW ratio of rats measured 48 h after LPS administration

LPS did not cause a significant loss of body weight (Table 2), but significantly increased the weight of the liver (*p* < .01) and the previous therapy with CDCA prevented this LPS-induced effect (*p* < .05). Ten-day pretreatment with UDCA and CDCA attenuated changes in the LW/BW ratio in LPS-induced endotoxemia.

**Table 2:** Effects of UDCA and CDCA pretreatment on changes in BW, LW, and LW/BW in rats

Parameters	Groups (mean ± SD)							
	C	LPS	UDCA		UDCA + LPS	CDCA		CDCA + LPS
BW (g)	218.33 ± 7.20	212.00 ± 13.25	228.00 ± 13.61	207.50 ± 17.62		218.83 ± 16.95	206.00 ± 10.54	
LW (g)	6.59 ± 0.32	10.92 ± 1.02††	6.43 ± 0.32	9.35 ± 1.42		6.00 ± 0.44	9.62 ± 0.53*	
LW/BW	0.030 ± 0.002	0.052 ± 0.003††	0.028 ± 0.001	0.045 ± 0.003*		0.027 ± 0.002	0.047 ± 0.002*	

Data are expressed as the mean ± SD. BW- body weight; LW- liver weight; LW/BW- liver to body weight ratio; C- control group; LPS- LPS treated group; UDCA- Ursodeoxycholic acid treated group; UDCA + LPS- Ursodeoxycholic acid and LPS treated group; CDCA- Chenodeoxycholic acid treated group; CDCA + LPS- Chenodeoxycholic acid and LPS treated group. Mann-Whitney test and asterisk (*) indicate significant differences compared with LPS group * *p* < .05 and sign (†) indicate significant differences compared with C group † *p* < .05, †† *p* < .01

### Effects of UDCA and CDCA pretreatment on biochemical parameters measured 48 h after LPS administration

AST, ALT, and LDH were used as markers of acute liver injury. The increased activity of AST and LDH in the LPS-treated group (*p* < .01) indicated that LPS had induced liver damage (Table 3). The activity of LDH was reduced in the UDCA + LPS group compared to the LPS group, demonstrating that UDCA mitigated the effects of LPS. A significant increase in blood glucose levels, CK, TnI, and Hcy was observed in all rats treated with LPS (Table 3). UDCA and CDCA did not affect the glucose levels in rats treated with LPS. UDCA made a significant decrease in TnI and CK (*p* < .01). Both UDCA and CDCA exhibited a significant decrease in Hcy (*p* < .001) (Table 3).

**Table 3:** Biochemical parameters of rats treated with UDCA, CDCA, and LPS

Parameters		Groups					
		C	LPS	UDCA	UDCA + LPS	CDCA	CDCA + LPS
Glucose (mmol/L)	12.70 ± 2.37		17.70 ± 4.37††	9.02 ± 1.76	16.61 ± 3.22	8.35 ± 2.18	16.92 ± 2.55
CK (U/L)	274.83 ± 40.01		5467.33 ± 1346.34††	627.27 ± 846.87	3514.67 ± 319.02*	1641.67 ± 1194.92	3038.03 ± 1648.39
AST (U/L)	100.83 ± 18.38		179.5 ± 71.73††	142.17 ± 28.06	154.67 ± 29.28	119.0 ± 30.49	150.00 ± 45.17
ALT (U/L)	71.67 ± 13.49		60.33 ± 22.39	59.33 ± 17.99	64.50 ± 21.24	64.00 ± 23.95	67.67 ± 17.12
LDH (U/L)	120.25 ± 56.67		955.33 ± 567.24††	187.16 ± 152.16	591.33 ± 143.25*	446 ± 270.92	899.00 ± 768.67
Troponin I (pg/mL)	8.22 ± 2.13		151.28 ± 48.14†††	14.62 ± 3.29	46.46 ± 8.75*	45.40 ± 17.47†	121.15 ± 66.58
Hcy (µmol/L)	4.78 ± 0.83		7.96 ± 0.71†††	5.83 ± 1.14	5.21 ± 0.70***	6.35 ± 1.39	4.58 ± 1.30***

Data are expressed as mean ± SD or mean ± SE. CK- creatine kinase; AST- aspartate aminotransferase; ALT- alanine aminotransferase; LDH- lactate dehydrogenase; Hcy- homocysteine; C- control group; LPS- LPS treated group; UDCA- Ursodeoxycholic acid treated group; UDCA + LPS- Ursodeoxycholic acid and LPS treated group; CDCA- Chenodeoxycholic acid treated group; CDCA + LPS- Chenodeoxycholic acid and LPS treated group. One-way ANOVA, Bonferroni test, and Mann-Whitney test were done, and asterisk (*) indicates significant differences compared with LPS group * *p* < .05, ** *p* < .01 *** *p* < .001, and sign (†) indicates significant differences compared with C group †† *p* < .01, ††† *p* < .001

The lipid profile was analyzed to investigate the effects of UDCA and CDCA on lipid metabolism. An increase of TC, LDL-C, and TG was observed in the LPS group (*p* < .01) compared to C group (Table 4). Pretreatment with UDCA led to a significant decrease in TG levels (*p* < .05), and pretreatment with CDCA resulted in a significant decrease in TG and LDL-C levels ( *p* < .05) (Table 4).

**Table 4:** Serum lipid profile of rats treated with UDCA, CDCA, and LPS

Parameters	Groups (mean ± SD)					
	C	LPS	UDCA	UDCA + LPS	CDCA	CDCA + LPS
TC (mmol/L)	2.13 ± 0.19	2.70 ± 0.39††	2.56 ± 0.27†	2.53 ± 0.15	2.18 ± 0.26	2.38 ± 0.20
LDL-C (mmol/L)	0.28 ± 0.04	0.43 ± 0.08 ††	0.35 ± 0.05†	0.35 ± 0.05	0.31 ± 0.04	0.30 ± 0.06*
HDL-C (mmol/L)	0.83 ± 0.08	0.85 ± 0.54	0.88 ± 0.11	0.80 ± 0.12	0.75 ± 0.10	0.80 ± 0.09
TG (mmol/L)	0.50 ± 0.06	0.88 ± 0.22††	0.48 ± 0.13	0.67 ± 0.05*	0.48 ± 0.08	0.65 ± 0.10*

Data are expressed as the mean ± SD. TC- total cholesterol; LDL-C- low-density lipoprotein cholesterol; HDL-C - high-density lipoprotein cholesterol; TG- triglycerides; C- control group; LPS- LPS treated group; UDCA- Ursodeoxycholic acid treated group; UDCA + LPS- Ursodeoxycholic acid and LPS treated group; CDCA- Chenodeoxycholic acid treated group; CDCA + LPS- Chenodeoxycholic acid and LPS treated group. One-way ANOVA, Bonferroni test, and Mann-Whitney test were done, and asterisk (*) indicates significant differences compared with LPS group * *p* < .05 and sign (†) indicates significant differences compared with C group † *p* < .05; †† *p* < .01

### Effects of UDCA and CDCA pretreatment on endothelial dysfunction measured 48 h after LPS administration

**Fig. 1:**
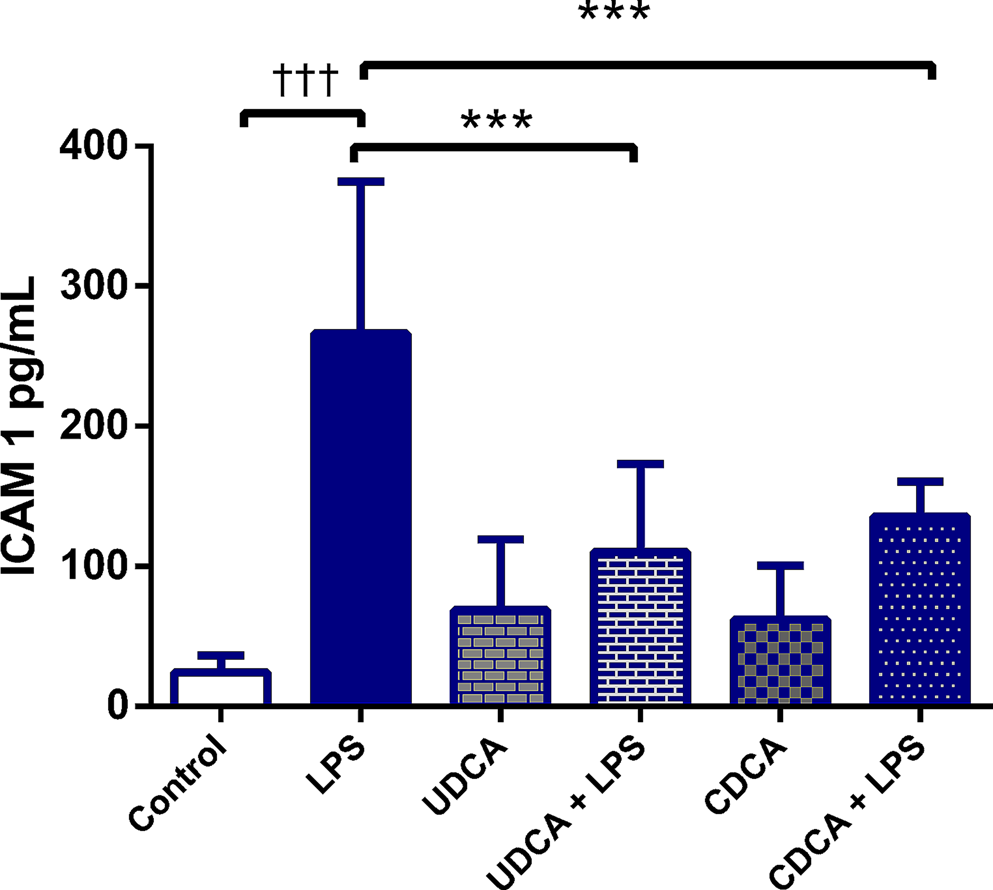
Effect of UDCA and CDCA pretreatment on the level of soluble ICAM-1 in plasma. C- control group; LPS- LPS treated group; UDCA- Ursodeoxycholic acid treated group; UDCA + LPS- Ursodeoxycholic acid and LPS treated group; CDCA- Chenodeoxycholic acid treated group; CDCA + LPS- Chenodeoxycholic acid and LPS treated group. One-way ANOVA, Bonferroni test, and Mann-Whitney test were done, and asterisk (*) indicates significant differences compared with LPS group *** *p* < .001, and sign (†) indicates significant differences compared with C group †††*p* < .001

Soluble ICAM-1 was used as a marker of endothelial dysfunction. ICAM-1 levels in the LPS group were significantly increased compared to the control (Fig. 1). The levels of ICAM-1 were decreased in the UDCA + LPS and CDCA + LPS group compared to the LPS group (*p* < .001) (Fig. 1).

### Effects of UDCA and CDCA pretreatment on oxidative stress markers 48 h after LPS administration

**Fig. 2:**
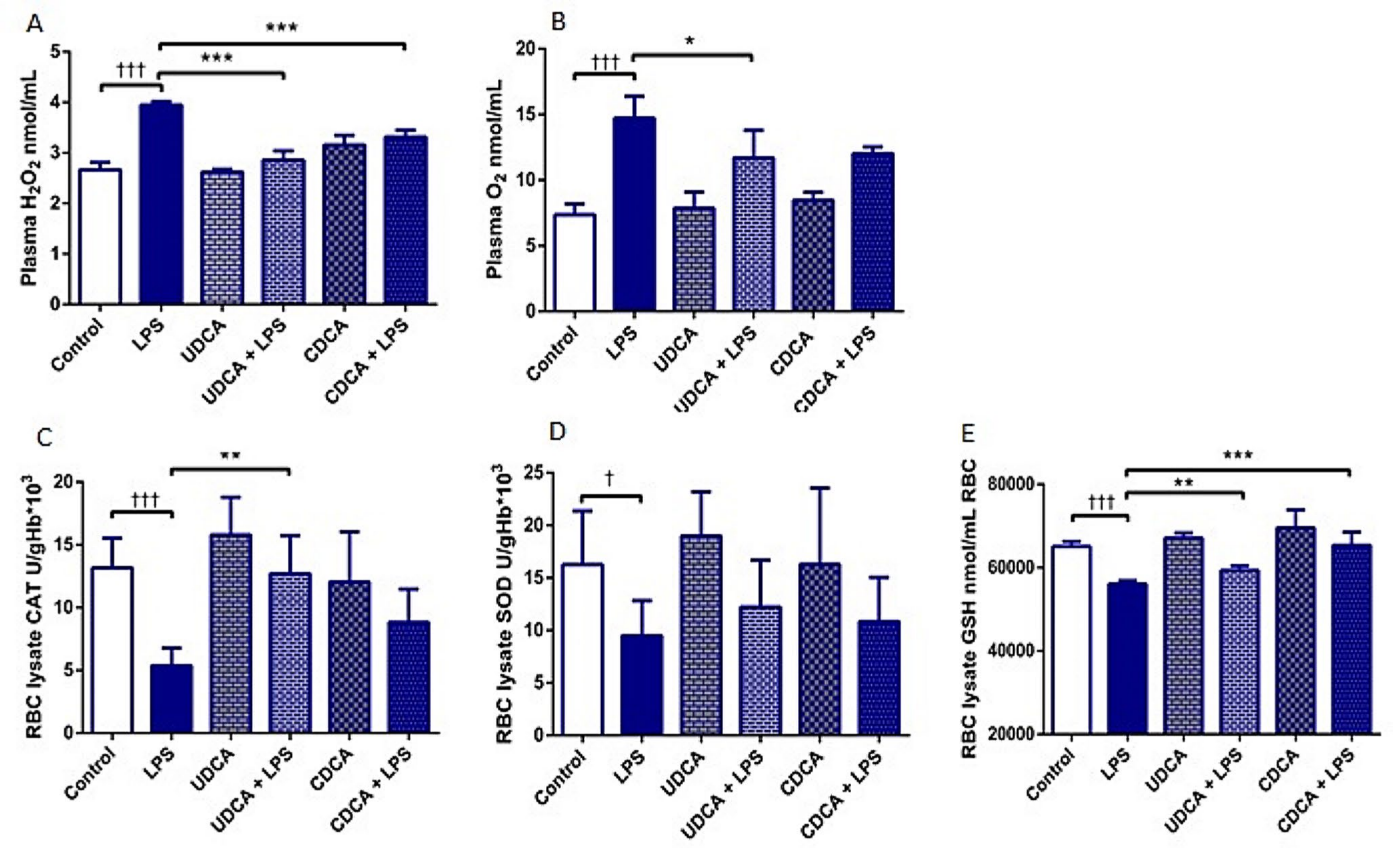
Effects of UDCA and CDCA on oxidative stress markers **(A)** Plasma H_2_O_2_ levels; **(B)** Plasma O_2_^-^ levels; and antioxidative enzymes activity **(C)** CAT activity in erythrocyte lysate; **(D)** Activity of SOD in erythrocyte lysate; **(E)** GSH level in erythrocyte lysate. Data are expressed as the mean ± SD or mean ± SE. C- control group; LPS- LPS treated group; UDCA- Ursodeoxycholic acid treated group; UDCA + LPS- Ursodeoxycholic acid and LPS treated group; CDCA- Chenodeoxycholic acid treated group; CDCA + LPS- Chenodeoxycholic acid and LPS treated group. One-way ANOVA, Bonferroni test, and Mann-Whitney test were done, and asterisk (*) indicates significant differences compared with LPS group * *p* < .05, ***p < .001 and sign (†) indicate significant differences compared with C group † < p 0.05; †† <0.01; ††† *p* < .001

UDCA and CDCA demonstrated antioxidative effects in LPS-induced endotoxemia (Fig. 2). ROS plays a crucial role in oxidative stress and, as representatives of ROS, were analyzed H_2_O_2_ and O_2_^–^. Levels of H_2_O_2_ and O_2_^–^ increased in the LPS group, however administration of UDCA and CDCA decreased the ROS production in LPS-induced endotoxemia (Fig. 2A and B). SOD, CAT, and GSH were examined as components of the antioxidative defence system. CAT, GSH, and SOD showed a decrease in the LPS-treated group (*p* < .001, *p* < .001 and *p* < .05, respectively) (Fig. 2). UDCA and CDCA once again demonstrated their beneficial effects on the activity of CAT and GSH levels (both *p* < .01) (Fig. 2C and E), with CDCA also showing a beneficial effect on the activity of GSH (*p* < .001) (Fig. 2E).

### Effects of UDCA and CDCA pretreatment on NF-κB expression in rat hepatocytes after LPS administration

**Fig. 3:**
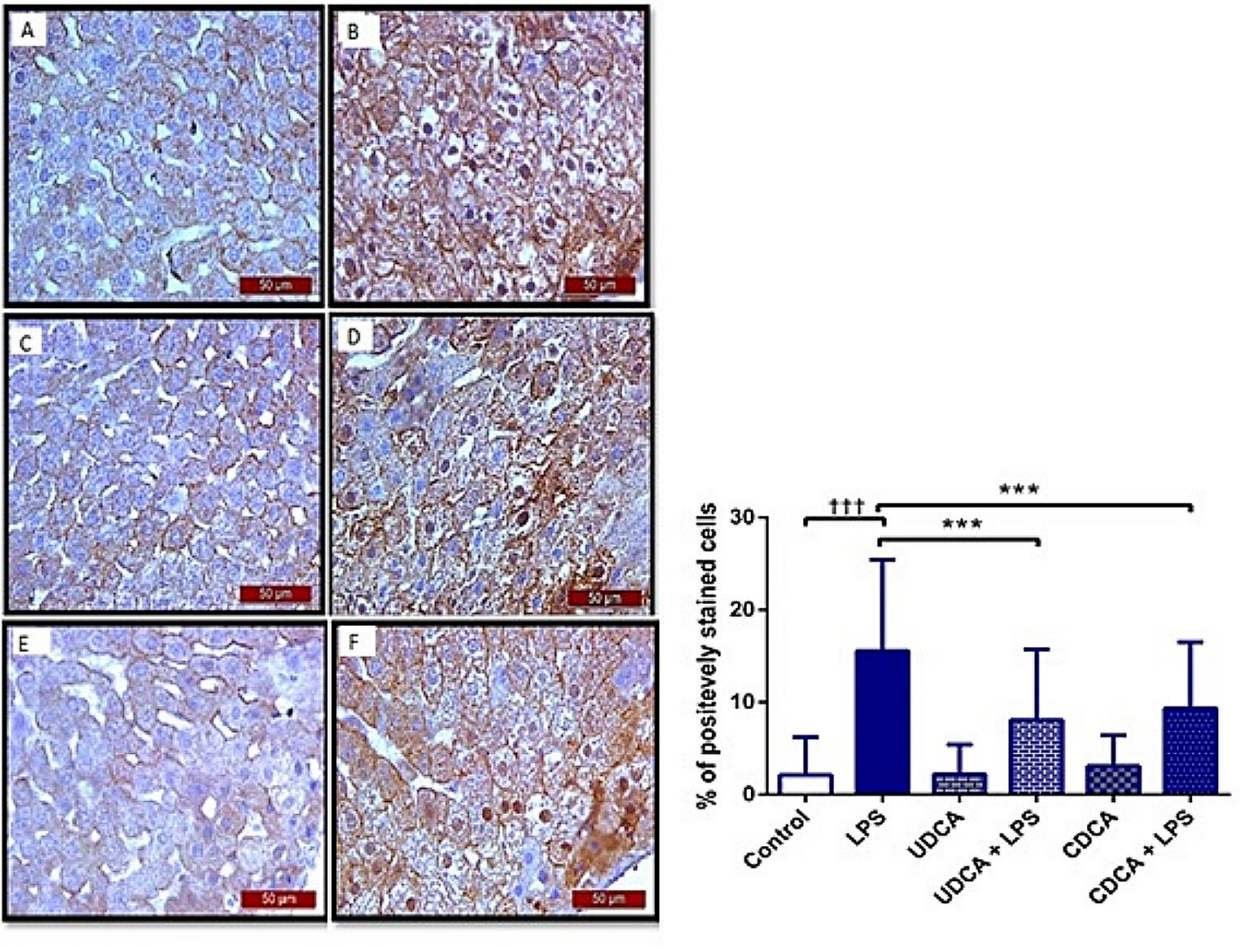
Immunohistochemical assessment of NF-kB in rat liver tissue (400x magnification): **(A)** Control group displayed no NF-kB expression. **(B)** The LPS group displayed a notable increase in NF-κB immunoreactivity within hepatocytes (nuclear staining). The brown color indicates NF-κB positivity (indicated by black arrows). **(C)** UDCA group revealed no NF-kB expression. **(D)** UDCA + LPS group illustrated a significant decrease in NF-κB immunostaining. **(E)** CDCA group also displayed no NF-kB expression. **(F)** CDCA + LPS group showed a marked reduction in NF-κB immunostaining. **(G)** % of positively stained cells. One-way ANOVA, Bonferroni test, and Mann-Whitney test were done, and asterisk (*) indicates significant differences compared with LPS group ***p < .001 and sign (†) indicates significant differences compared with C group ††† *p* < .001

Our findings indicate that the immunohistochemical analysis of rat liver (Fig. 3A-F) revealed a substantial elevation in NF-κB immunoreactivity in hepatocytes following LPS administration compared to the control group (Fig. 3G, *p* < .001). Conversely, rats pretreated with UDCA and CDCA exhibited a significant reduction in the LPS-induced overexpression of NF-κB in hepatocytes (Fig. 3G, *p* < .001). Rats receiving UDCA or CDCA alone displayed negative immunostaining for NF-kB in liver tissue, resembling the pattern observed in the control group. These results strongly suggest a significant attenuation of NF-κB expression in LPS-induced endotoxemia through the administration of UDCA and CDCA.

### Histological analysis

**Fig. 4:**
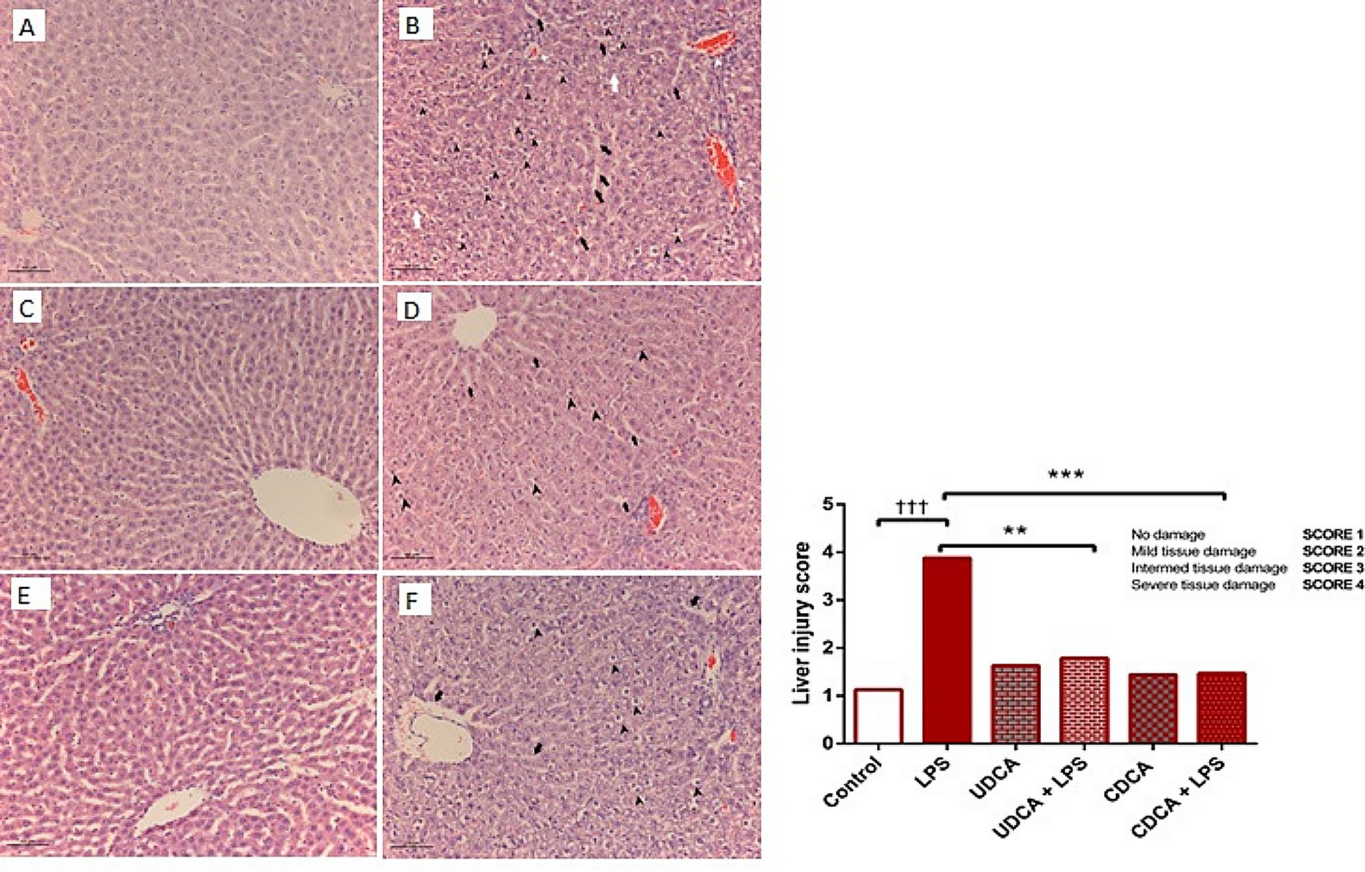
Representative histopathological examination of hematoxylin-and-eosin-stained photomicrographs of rat livers: **(A)** Control group. A light photomicrograph showing normal liver tissue. **(B)** LPS group. A light microphotograph displays the loss of lobular architecture and the dissociation of hepatocyte plates (indicated by white arrows). Additionally, mild sinusoidal dilatation and edema in the perisinusoidal space are visible (indicated by black arrows). Hepatocytes exhibit ballooning degeneration and multifocal necrotic cells are evident (indicated by black arrowheads). Congestion is observed in both the central vein and portal blood vessels (indicated by white arrowheads). **(C)** UDCA group. A light photomicrograph depicts normal liver tissue with mild sinusoidal dilatation. **(D)** UDCA + LPS group. A light photomicrograph depicts liver tissue with partially salvaged lobular architecture, featuring mild sinusoidal dilatation (indicated by black arrows). Hepatocyte plates maintain their normal structure, and ballooning degeneration of the cells is only evident in zone II (indicated by black arrowheads). Zone I and zone III hepatocytes exhibit normal nucleus and cytoplasm. **(E)** CDCA group. A light photomicrograph displays normal liver tissue with mild sinusoidal dilatation and extravasation in perisinusoidal spaces. (H&Ex200). **(F)** CDCA + LPS group. A light photomicrograph illustrates liver tissue with partially preserved lobular architecture, accompanied by mild perivascular and sinusoidal edema (indicated by black arrows). In addition, some Zone I and Zone II hepatocytes exhibit vacuolation (indicated by black arrowheads), whereas Zone III hepatocytes maintain a normal nucleus and cytoplasmic appearance. (H&Ex200). **(G)** Liver injury score. The liver injury score was calculated based on the HE staining images

Microscopically, the liver tissues of control animals exhibited normal histology (Fig. 4A). In rats treated with LPS alone, significant hepatic injury was seen. Light microphotographs displayed a loss of lobular architecture and hepatocyte plate dissociation. Mild sinusoidal dilatation and perisinusoidal space edema were evident. Hepatocytes exhibited ballooning degeneration with multifocal necrotic cells present. Congestion was observed in the central vein and portal blood vessels (Fig. 4B). In rats treated with UDCA alone, photomicrographs showed normal liver tissue with mild dilatation and extravasation in perisinusoidal spaces (Fig. 4C). In the LPS/UDCA group of rats exhibited liver tissue with partially salvaged lobular architecture, mild sinusoidal dilatation, and ballooning degeneration in zone II hepatocytes. Zone I and zone III hepatocytes displayed normal nucleus and cytoplasm (Fig. 4D). In rats treated with CDCA alone, light photomicrographs displayed normal liver tissue with mild sinusoidal dilatation and extravasation in perisinusoidal spaces (Fig. 4E). In the LPS/CDCA group of rats, our results showed liver tissue with partially salvaged lobular architecture and mild perivascular and sinusoidal edema. Some Zone I and Zone II hepatocytes were vacuolated, while Zone III hepatocytes exhibited normal nucleus and cytoplasm (Fig. 4F). A semi-quantitative pathological score based on the severity of tissue damage is presented in Fig. 4G.

## Discussion

The findings of this study demonstrated the anti-inflammatory, antioxidative, and liver-protective effects of UDCA and CDCA in the LPS-induced model of inflammation in rats. This is based on the ability of these bile acids to reduce serum levels of pro-inflammatory cytokines, soluble ICAM-1, and liver enzymes, decrease prooxidative markers, increase antioxidative enzymatic activity, normalize serum lipid status, and to reduce the NF-κB expression in hepatocytes and mitigate liver injury.

The application of LPS triggers acute systemic inflammation by activating the immune system via its binding to a receptor complex composed of CD14, TLR4, and myeloid differentiation factor-2 (MD-2) [31]. The signaling through TLR4 upon binding of LPS is transmitted by two cascades: the first one involves adaptor proteins myeloid differentiation primary response gene 88 (MyD88) and Toll/IL-1 receptor (TIR) adaptor protein (TIRAP) in the plasma membrane; the second one engages adaptor proteins TIR domain-containing adaptor-inducing interferon-beta (TRIF) and TRIF-related adaptor molecule (TRAM) in early endosomes after endocytosis of the receptor complex. The MyD88-dependent pathway includes the activation of NF-κB, by dissociating its inhibitory subunit, and subsequent translocation of the activated form to the nucleus where it activates numerous genes coding pro-inflammatory cytokines, chemokines, growth factors, and immune activation biomolecules. The TLR4/TRIF pathway induces the phosphorylation of interferon-regulatory factor-3 (IRF-3) followed by the activation of genes coding IFN-β. These signaling pathways are competitive and mutually exclusive [32]. An early effect of LPS on diverse cell types, including neutrophils, macrophages, mast cells, platelets, and endothelial cells is characterized by the production of IL-1 and TNF-α. These cytokines play a crucial role in promoting the pro-inflammatory state, triggering the release of additional cytokines like IL-6, IL-8, GM-CSF, INF-γ, and other cytokines of both innate and adaptive immunity [33]. The results from our previous study clearly showed that a non-lethal dose of LPS induced a significant increase in concentrations of TNF-α, IL-1β, and IL-6 within the first 2 h, followed by a slow and sustained decrease in subsequent hours [34]. These results are confirmed in this study and extended to GM-CSF, IL-2, and IFN-γ.

Numerous investigations showed that UDCA has an anti-inflammatory effect [11–13, 20], suggesting the benefits of this bile acid therapy in acute inflammatory response. A similar effect was published for CDCA as well [21–23, 35], but some studies suggested its pro-inflammatory role in promoting liver injury [24, 25]. Our results demonstrated that both bile acids can attenuate the production of IL-1β, TNF-α, IL-6, GM-CSF, and IFNγ induced by the LPS administration, suggesting its potential beneficial effect in suppressing systemic inflammation. At the same time, these results suggested that NF-κB could be a key target of their effect. However, other targets could be also involved such as Akt, ERK, JNK, and p38 [20]. Some differences observed (suppression of IFN-γ by CDCA or suppression of IL-6 by UDCA) could be the consequence of interindividual variability between the groups or different affinity of these bile acids to their receptors as shown for FXR and TGR5 [23]. Although UDCA and CDCA have different structures, they can act on different receptors and signaling molecules in a given immune cell, and therefore, the outcome may be the result of competition among different receptors and generated signals [36, 37]. In this context, CDCA has been shown to inhibit autophagy in an FXR-dependent mechanism, whereas UDCA stimulates the formation of autophagosomes independently of FXR and enhances autophagic flux [38]. Neither CDCA nor UDCA exerted any significant effects on the production of pro-inflammatory cytokines when applied alone, suggesting that these bile acids could be safe under physiological conditions.

Hepatomegaly, often characterized by an increase in liver weight, is a common observation in sepsis, and it can be attributed to various pathophysiological mechanisms. The liver weight-to-body weight ratio serves as an indicator of hepatomegaly and functions as a marker for liver disease [39]. UDCA, as a secondary bile acid, is known for its reduced cytotoxicity compared to primary bile acids synthesized in the liver [40]. The anti-inflammatory and antioxidant properties of UDCA and CDCA play a role in preserving liver function, reducing hepatomegaly, and enhancing the overall prognosis of sepsis [41]. The results of this study showed an increase in LW and LW/BW ratio in the LPS-treated group, indicating that LPS caused liver enlargement. In the current study, UDCA and CDCA pretreatment decreased the LW/BW ratio, and CDCA also reduced the LW, thereby preventing LPS-induced liver enlargement.

The presence of LPS in the cell wall of Gram-negative bacteria initiates an inflammatory response, leading to significant alterations in glucose metabolism. Hyperglycaemia is commonly detected in bacterial infections and serves as an indicator of unfavorable clinical prognosis [42]. Bile acids possess hormone-like properties in regulating both, lipid and glucose metabolism. The administration of UDCA resulted in reduced glucose levels, elevated serum GLP-1 levels, and relief from hyperinsulinemia [43]. However, our results showed that UDCA and CDCA did not affect the glucose levels in rats treated with LPS. The results of the most recent clinical study, conducted at our research centre, indicated that the eight weeks administration of UDCA tablets did not lead to a significant decrease in fasting blood glucose levels among patients with type 2 diabetes mellitus, but significantly attenuated oxidative stress parameters, body mass index, and liver enzymes [44].

Sepsis is frequently linked with rhabdomyolysis, and Gram-positive bacterial pathogens are commonly cited as the leading cause of sepsis-induced rhabdomyolysis. The key clinical indicator in the development of rhabdomyolysis is the elevation of creatine kinase (CK) levels [45]. In this study, we observed an increase in CK levels in the LPS group, and this increase was mitigated by the administration of UDCA. The reasons for the increase in CK levels after treatment with UDCA and CDCA (without the application of LPS) could be consequences of the muscle response to the administration of UDCA and CDCA, which may impact muscle cells and lead to an increase in CK as a response to changes in muscle tissue, which does not necessarily imply the presence of rhabdomyolysis. Additionally, the increase in CK may be associated with individual sensitivity to UDCA and CDCA.

In cases of toxic injury, hepatocyte necrosis leads to the release of enzymes into the bloodstream. AST and ALT are the most frequently utilized indicators of hepatocyte damage. In comparison to AST and ALT, lactate dehydrogenase (LDH) is a less specific marker for hepatocyte injury [47]. The AST, ALT, and LDH levels increased after LPS injection [48]. Our results showed a significant increase in AST and LDH levels in the LPS-treated group and a decrease of LDH in the UDCA-treated group, suggesting that UDCA mitigates the LPS-induced changes in LDH activity.

Limited-scale investigations have indicated that increased troponin levels can identify septic patients with an increased risk of mortality. Furthermore, patients with elevated troponin levels exhibited a higher mortality rate [49]. Individuals affected by sepsis often display increased levels of cardiac troponin I, even in the absence of coronary artery disease [50]. Our study demonstrated a noteworthy rise in hsTnI levels in rats treated with LPS, which were mitigated by UDCA. However, CDCA did not result in a reduction of CK, LDH, and hsTnI levels. The rationale behind these results could be found in the structural composition of bile acids, where the cytotoxicity is influenced by their specific molecular arrangements. Notably, UDCA and CDCA represent structurally distinct bile acids. UDCA, resulting from the dehydroxylation of free CDCA, emerges as the least toxic among them.

Homocysteine has been identified as a pro-inflammatory compound that can stimulate the production of specific cytokines, potentially contributing to the development of cardiovascular disease [51]. Elevated levels of homocysteine in sepsis have been linked to higher mortality rates. Homocysteine plays a significant role in septic patients due to its pro-inflammatory and procoagulant effects [52]. The favorable impact of UDCA on dyslipidemia and cardiovascular disease risk can potentially be explained by several antioxidant mechanisms. UDCA treatment appears to act against oxidative damage dependent on iron and hydroxyl radicals, inhibits the products of lipid peroxidation, and prevents the oxidative stress induced by reactive oxygen species through the activation of the PI3K/Akt/Nrf2 pathway in hepatocytes [53]. Recent research has shown a notable increase in oxidative stress products with the thickening of arterial intima and an elevated peroxidative glutathione redox status has been associated with the progression of atherosclerosis [54]. The current investigation showed that LPS led to an increase in homocysteine levels, which was prevented by UDCA and CDCA in rats treated with LPS.

The lipid profile was assessed by analyzing TC, HDL, LDL, and TG levels. Rats treated with LPS displayed elevated levels of TC, LDL, and TG. These findings are in alignment with those of Brigatto et al. [55], except for the increase in HDL concentration. UDCA is known for its potential to ameliorate dyslipidemia and reduce the risk of atherosclerotic cardiovascular disease due to its antioxidant properties [53]. In the current investigation, both UDCA and CDCA prevented the impairment of the lipid profile induced by LPS.

Endothelial cells may also have different immune functions and play a pivotal role in the systemic response to bacterial infections, working to limit their spread. When exposed to pathogens and microbial toxins, the responses of endothelial cells are diverse, heterogeneous, and multifaceted. During sepsis, endothelial cells transform into a proapoptotic, pro-inflammatory, proadhesive, and procoagulant phenotype. Additionally, damage to the glycocalyx and impaired vascular tone disrupt microcirculatory blood flow, leading to organ damage and the potential for organ failure [56]. In endothelial cells, UDCA counters endothelial dysfunction by inhibiting endoplasmic reticulum stress, reducing the expression of the receptor for advanced glycation end products, dampening the inflammatory response (including NF-κB activation), and suppressing the production of ROS. These effects are particularly valuable under hyperglycemic conditions, which are also present in sepsis [16]. Our findings indicate that UDCA and CDCA pretreatment reduced the level of ICAM thus ameliorating endothelial disfunction in the context of LPS-induced endotoxemia.

The presence of oxidative stress in LPS-induced endotoxemia has been already documented [57–59]. UDCA mitigates oxidative stress by acting as a molecule that scavenges ROS and reinforces the endogenous antioxidant defense [14]. Our study revealed a reduction in intracellular ROS levels, which had been elevated due to LPS, following treatment with UDCA and CDCA. We analyzed prooxidative markers and the antioxidative potential of UDCA and CDCA. The findings confirmed that LPS induced a significant level of oxidative stress, as indicated by the increased levels of H_2_O_2_ and O_2_^−^. In the current study, UDCA decreased the levels of H_2_O_2_ and O_2_^−^, while CDCA solely decreased the H_2_O_2_ levels. We assessed antioxidative defense by examining the activity of CAT, SOD, and GSH levels. The activity of CAT and SOD, as well as the level of GSH, were found to be decreased in LPS-induced endotoxemia, aligning with the results of several previous studies [19, 60]. Notably, both UDCA and CDCA significantly elevated the activity of CAT and GSH levels, thus increasing their antioxidative effects in the context of endotoxemia.

LPS is widely recognized as a stimulator of MAPKs, specifically the ERK, JNK, and p38 signaling pathways, which play crucial roles in regulating inflammatory responses. These pathways undergo phosphorylation in response to LPS. Additionally, NF-κB serves as a key regulator in pro-inflammatory signaling pathways. Initially present in the cytoplasm in an inactive form along with the inhibitory protein IκBα, NF-κB undergoes translocation to the nucleus for downstream inflammatory processes when IκBα is phosphorylated by inflammatory stimuli [20]. UDCA inhibits NF-κB by functionally modulating both the glucocorticoid receptor and the transcription processes dependent on NF-κB. Given the genes and proteins associated with NF-kB-dependent gene and the inactivator for NF-κB (IκBα), the reported observations propose that UDCA contributes to suppressing the production of pro-inflammatory cytokines and NO by deactivating NF-κB [61]. Certain studies have suggested that UDCA has the potential to hinder the activation of NF-κB and mitigate the phosphorylation of ERK, JNK, and p38 signals associated with inflammatory pathways [62]. A similar effect was published for CDCA as well [63]. In the present study, exposure to LPS resulted in a notable elevation in NF-κB immunoreactivity within liver tissue, and UDCA and CDCA exhibited a significant decrease in the LPS-induced overexpression of NF-κB in the liver tissue.

Pathohistological analysis revealed the normal homogenous structure of liver tissue in the control group, unlike the LPS-treated group where the liver lobule architecture, vascular congestion, hemorrhage, extravasation, determination of hepatocyte necrosis, and the presence of inflammatory cells were observed. Our results showed that the UDCA and CDCA protect liver cells from damage caused by LPS. At the same time, the administration of LPS alone leads to a significant liver injury, with changes in the structure of liver cells and sinusoids.

In numerous studies, UDCA has consistently exhibited its cytoprotective effects. Conversely, while CDCA has also been investigated for its cytoprotective activities (22, 24), the literature describes data on its cytotoxic effects. Studies have indicated that CDCA can induce endothelial activation through the stimulation of NF-kB and p38MAPK signalling pathways, likely mediated by the induction of reactive oxygen species (ROS) [65]. Moreover, CDCA has been shown to enhance the release of IL-6, TNF-α, and vascular endothelial growth factor (26), underscoring the importance of comprehensively understanding the effects of these acids on cellular health. Consequently, further research is warranted to reconcile the conflicting information regarding the actions of CDCA and to precisely delineate its role.

In conclusion, our experimental study indicates that prior administration of UDCA and CDCA offers advantageous outcomes in mitigating LPS-induced endotoxemia in rats. Our findings suggest that UDCA and CDCA, which are structurally distinct bile acids and exert slightly different effects, could both potentially play a role in preventing severe conditions associated with endotoxemia. This hypothesis is based on the findings that pretreatment with UDCA and CDCA reduces the levels of serum pro-inflammatory cytokines and NF-κB expression in hepatocytes, counteracts alterations in LW/BW ratio, prevents liver injury, improves lipid profiles, reduces oxidative stress and enhance antioxidative enzyme capacity.

## Data Availability

The authors will provide access to the dataset underpinning the findings of this research.
